# A hybrid microfluidic-vacuum device for direct interfacing with conventional cell culture methods

**DOI:** 10.1186/1472-6750-7-60

**Published:** 2007-09-20

**Authors:** Bong Geun Chung, Jeong Won Park, Jia Sheng Hu, Carlos Huang, Edwin S Monuki, Noo Li Jeon

**Affiliations:** 1 Department of Biomedical Engineering, University of California Irvine, Irvine, CA, 92697, USA; 2 Department of Pathology & Laboratory Medicine, University of California Irvine, Irvine, CA, 92697, USA

## Abstract

**Background:**

Microfluidics is an enabling technology with a number of advantages over traditional tissue culture methods when precise control of cellular microenvironment is required. However, there are a number of practical and technical limitations that impede wider implementation in routine biomedical research. Specialized equipment and protocols required for fabrication and setting up microfluidic experiments present hurdles for routine use by most biology laboratories.

**Results:**

We have developed and validated a novel microfluidic device that can directly interface with conventional tissue culture methods to generate and maintain controlled soluble environments in a Petri dish. It incorporates separate sets of fluidic channels and vacuum networks on a single device that allows reversible application of microfluidic gradients onto wet cell culture surfaces. Stable, precise concentration gradients of soluble factors were generated using simple microfluidic channels that were attached to a perfusion system. We successfully demonstrated real-time optical live/dead cell imaging of neural stem cells exposed to a hydrogen peroxide gradient and chemotaxis of metastatic breast cancer cells in a growth factor gradient.

**Conclusion:**

This paper describes the design and application of a versatile microfluidic device that can directly interface with conventional cell culture methods. This platform provides a simple yet versatile tool for incorporating the advantages of a microfluidic approach to biological assays without changing established tissue culture protocols.

## Background

Gradients of chemokines and growth factors play an important role in many developmental and physiological processes such as axon guidance [1], immune response [2], morphogenesis [3], and cancer metastasis [4,5]. Traditionally, Boyden chamber [6], under-agarose assay [7], and micropipette-based assay [8] have been used to study cellular behavior in soluble gradients. Unfortunately, these conventional assays are not capable of generating and maintaining stable gradients over long periods due to inherent limitations of their macroscale approach [6,7]. In addition, they typically require relatively large amounts of costly reagents and often do not allow real-time monitoring of cell behavior.

Recent developments in microfluidic devices that apply miniaturization technologies from the microelectronics industry have resulted in microscale devices that hold the promise of overcoming limitations imposed by conventional macroscale methods. Microfluidic devices that can generate stable soluble and surface gradients of complex profiles have been developed in last few years [9-11]. These gradient-generating microfluidic devices allow miniaturized real-time cellular assays [12-14] for investigating migration [15], proliferation, differentiation [16], and apoptosis [17]. We have previously reported on "Christmas-tree" network-based microfluidic devices for investigating migration of neutrophils [18,19] and metastatic cancer cells [20,21] as well as differentiation of human neural progenitor cells (NPCs) [16]. Although these devices can create stable concentration gradients, one important limitation has been the need to culture cells inside the microfludic devices before each experiment. Setting up a microfluidic experiment required at least 2–3 hours of preparation time for each set resulting in throughput of 2–3 experiments a day. Furthermore, it was difficult to perform experiments with sensitive cells such as NPCs and neurons that did not plate well in the devices.

Recently, a network of vacuum channels was used to provide uniform bonding to any underlying flat surface [22]. This vacuum-sealed microfluidic device was used to investigate the kinetics of leukocyte adhesion under defined shear flow in 6-well tissue culture plates. Here we developed a novel hybrid microfluidic-vacuum device that incorporates a network of vacuum channels and a microfluidic gradient generator. Unlike the previous vacuum-sealed device [22], which generates differential shear stress, our hybrid microfluidic-vacuum device generates concentration gradients of soluble factors in a single chip. This device has several advantages over previous microfluidic devices that required bonding of two separate pieces: (i) it allows the reversible application of microfluidic gradients onto wet cell culture surfaces, (ii) it demonstrates high reproducibility and functionality, and (iii) it is a highly adaptable method for incorporating the advantages of microfluidics to biological assays without changing well established tissue culture protocols. This hybrid device is a versatile experimental platform that is compatible with various wet biology protocols and should find a number of applications in biomedical research.

## Results and discussion

### Design of the hybrid microfluidic-vacuum platform for gradient generation

**Figure 1:**
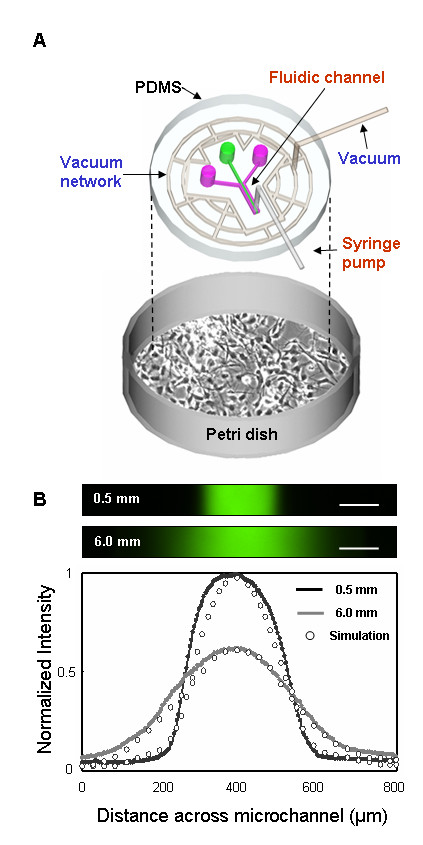
A hybrid microfluidic-vacuum platform for interfacing with Petri dish. (A) Schematic design of the microfluidic device that show separate vacuum and fluidic channels. Side reservoirs (purple) contain buffer while buffer with FITC-Dextran (green) filled the middle reservoir. Parallel laminar flows from the three reservoirs generate stable gradients by diffusion. (B) Gradients of varying slope and profile generated in a single device. Two different gradient profiles generated at 0.5 mm and 6 mm downstream of the junction were visualized by FITC-Dextran profile. Gradient profiles were generated using 1 μl/min withdrawal rate. Experimentally measured and calculated gradient profiles were normalized for comparison. Scale bars are 100 μm.

Figure 1A is a schematic of the microfluidic-vacuum device. Instead of irreversible bonding to create enclosed channels, we used a hybrid design that incorporated separate channels for vacuum and liquids. The vacuum channels were used for reversible bonding to a hard surface. An important consideration was to place the fluidic channels in the center of the device and surround it with a network of interconnected vacuum channels around the periphery. For these studies, we incorporated a simple microfluidic gradient generator composed of three inlet reservoirs and one outlet. For fluid flow, a syringe pump connected to channel outlet was operated in withdrawal mode to minimize pressurizing the device, which disrupted the seal between device and culture surface.

There were no significant design limitations for vacuum channels; a wide range of dimensions (50~500 μm wide) and designs (nested squares and circles) were successfully used. The vacuum channels were placed anywhere between 0.2–2 mm away from the fluidic channels. When the channels were placed closer, chance for leakage between vacuum channels and fluidic channels was greater, and it often led to failed seals. Due to the soft elastomeric property of poly(dimethylsiloxane) (PDMS), the vacuum channels provided dependable bonding of the hybrid device onto flat glass or plastic. Surface coatings (e.g. poly-L-lysine, laminin, and collagen) had no obvious effects on vacuum bonding or on subsequent gradient generation. In addition, we measured the vacuum force on a tip of tubing connected with a syringe to the house vacuum line. The vacuum pressure required to maintain the seal was 1–10 torr (this value may vary depending on device design and other experimental variables). Use of house vacuum was sufficient for the experiments to work reproducibly.

By flowing different solutions from the three inlet reservoirs, continuous laminar streams can generate stable gradients that are perpendicular to the flow. Multiple gradient profiles were generated at different downstream positions of the inlets (e.g. a few mm apart) on a single device and controlled by adjusting flow rates (0.3–1 μl/min). Cells exposed to these flow rates in a gradient chamber were subjected to fluid shear stress in the range of 4.2 × 10^-3^-1.4 × 10^-2 ^dyne/cm^2^. This value is significantly lower (approx. 2–3 orders of magnitude) than physiological shear stress on endothelial cells during normal blood flow (~2 dyne/cm^2^) [23]. Figure 1B shows two different gradient profiles at 0.5 mm and 6 mm downstream from the junction of fluidic channels (1 μl/min withdrawal rate) containing fluorescein isothiocyanate (FITC)-dextran (10 μM, MW = 10 kDa) in the middle reservoir, while the side reservoirs contained buffer only. The gradients could be maintained for 6~24 hours depending on reservoir capacity (150~600 μl) and flow rate. Gradient profiles of FITC-dextran compared favorably to simulation results (Fig. 1B).

An important advantage of the hybrid microfluidic device is that cell-based experiments can be routinely performed due to simpler setup and shorter setup time compared to conventional microfluidic gradient devices. Moreover, cell cultures can be prescreened for optimal health, density, and distribution. Setup time was typically < 5 min, allowing for several time-lapse cell migration experiments per day. Microfluidic experiments could be initiated whenever cells were ready in Petri dish or multi-well culture plates (our studies were performed using 6-well plates). Other advantages are: (i) the hybrid device allows experiments with sensitive cells (e.g. mouse NPCs, neurons) that are difficult to culture inside microfluidic devices, and (ii) reversible bonding allows for straightforward immunocytochemistry or other endpoint analyses after gradient applications. To remove the device after an experiment, vacuum was turned off after adding about 2 ml of media (enough to completely immerse the device in a 6-well plate). After a few minutes, the device detached from the surface and floated to the surface. Attachment and viability of cells were not adversely affected by this release process.

Although this device brings many advantages to cell culture studies, a limitation is that the design is not compatible with complex fluidic networks. Long, serpentine channels have a higher chance of bonding failure. Bonding failure can occur if the surface is uneven (i.e. trapped large debris) or if there is a leak between the vacuum network and fluidic channels. Bonding failure rate was relatively low (< 5%) and could be further reduced by using simple fluidic channels and providing a sufficient gap (0.2–2 mm) between the vacuum network and fluidic channels. Device integrity and gradient quality were monitored by checking the fluorescence gradient profile in the microchannel by adding a small amount of fluorescent dye in one of the reservoirs.

### Real-time live and dead assay of mouse NPCs in a microfluidic device

Compared to human NPCs [16], we found that mouse NPCs were difficult to culture inside irreversibly-bonded microfluidic devices. Initial plating efficiency of mouse NPCs was poor, and they displayed lower proliferation rates and higher cell death rates compared to Petri dish controls (data not shown). We therefore cultured mouse NPCs in 6-well plates, then selected healthy cultures for vacuum device application.

**Figure 2:**
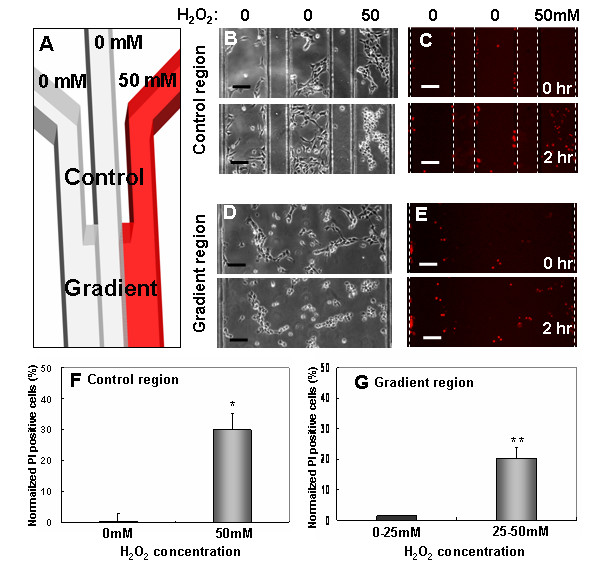
Real-time optical live/dead assay for mouse NPCs using microfluidics. (A) Schematic drawing of a fluidic network containing control and gradient region. (B-C) Mouse NPCs cultured in the control region of fluidic channel. Hydrogen peroxide (50 mM) was added into the right reservoir after preloading with propidium iodide (red). Cell death resulting from exposure to hydrogen peroxide was monitored by time-lapse microscopy. (D-E) Numbers of PI-positive cells increase towards the right side of the gradient chamber with longer exposure to hydrogen peroxide. Quantitative analysis of cell death in the control (F) and gradient region (G). Each bar shows the average for three independent experiments with standard errors of mean (*p < 0.05, **p < 0.01). Scale bars are 100 μm.

To test the ability of this device to seal and generate gradients, we exposed mouse NPCs to hydrogen peroxide (H_2_O_2_) gradients, a rapid inducer of cell death [24,25]. To simultaneously investigate cellular responses to fixed and graded hydrogen peroxide concentrations, the fluidic channel network contained both control and gradient regions and time-lapse images were taken in both regions. The control region had three microchannels that combined into a single channel where gradients were formed (Figure 2A). Cell-impermeant propidium iodide (PI) at 2 μg/ml was preloaded to identify dead cells before gradient application. After 2 hours of exposure to hydrogen peroxide (right inlet) with a flow rate of 0.3 μl/min, cells in the two left channels were alive and PI-negative (Figure 2B), while cells in the right channel became PI-positive (Figure 2C) [see Additional File 1, 2]. In the gradient region, an increasing number of cells showed PI staining over time and from right to left as hydrogen peroxide diffused from the right inlet channel and across the main channel (Figure 2D, E) [see Additional File 3, 4]. Cell death corresponded well to the concentration of hydrogen peroxide in both control and gradient chambers (Figure 2F, G). We divided the gradient region into two areas based on the mean concentration (25 mM) of hydrogen peroxide based on simulations. (Note: for most experiments, fluorescent gradient profiles of FITC-dextran with molecular weight similar to agonist can be used to monitor gradient profiles. This was not performed here, since the molecular weight of hydrogen peroxide is much smaller than fluorescent dyes).

During experimental setup, some cells become trapped at the edges of channels (Fig. 2B, C) and might interact with live healthy cells in the channels. These cell-cell interactions might lead to false positive results. However, the hybrid vacuum devices should minimize such results. First, due to continuous perfusion, paracrine signaling by trapped dead cells should be transient and minimal, particularly in the orthogonal direction. Second, one can avoid interpretive pitfalls by avoiding cells near the edges. Third, edge effects can be minimized by making the channels larger (lower edge/area ratio). These results confirmed the ability of vacuum to seal and isolate the microfluidic channels using live cells grown on a Petri dish.

### Cancer cell chemotaxis

One of the main applications for microfluidic gradients are studies on cell migration and chemotaxis [12-14]. To further compare the hybrid microfluidic-vacuum device to conventional microfluidic chemotaxis chambers, we performed chemotaxis assays with MDA-MB-231, a human metastatic breast cancer cell line. For our experiment, epidermal growth factor (EGF) was used as a chemoattractant, since it has been implicated as an important factor in breast cancer cell metastasis, and it was shown previously that MDA-MB-231 cells responded to EGF gradients in a chemotactic manner [20,21]. In general, linear gradients of EGF were not effective in inducing directional cell movement as compared to non-linear concentration gradients.

**Figure 3:**
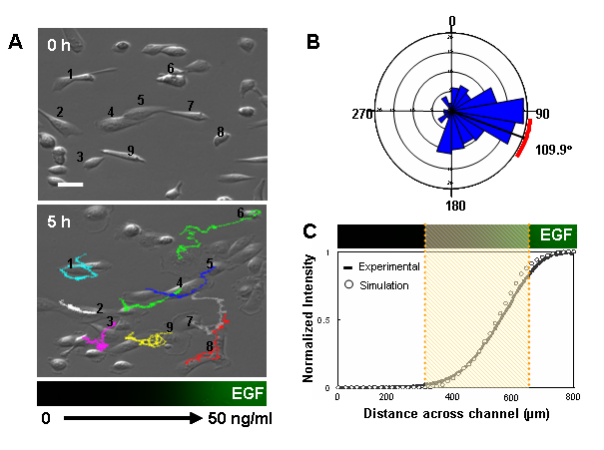
Chemotaxis of human breast cancer cells in a concentration gradient (0–50 ng/ml) of EGF. (A) First and last images of cells in the gradient region after 5 hours. (B) Directional plot of migration angle in 3–45 ng/ml EGF gradients, grouped in 10° intervals. (C) Cells inside the dotted lines (3–45 ng/ml EGF) were tracked and analyzed. The EGF gradient profile is visualized by FITC-dextran (10 kD) with a similar molecular weight to EGF. Scale bars are 20 μm.

Using the hybrid device, MDA-MB-231 cells were observed for 5 hours in an EGF gradient (0–50 ng/ml) with a withdrawal rate of 0.3 μl/min. EGF-containing media was added to the right reservoir while the other reservoirs contained media alone. Figure 3A shows that cells were observed migrating toward higher concentrations of EGF. Polarized extensions of lamellipodia toward higher EGF concentrations were observed in the majority of cells [see Additional File 5]. A Rayleigh test revealed that migration was directional toward high EGF concentration, and the mean migration angle was 109.9° (*p *< 0.01; Figure 3B). In addition, cells in the steepest part of the gradient (3–45 ng/ml) displayed the greatest directional migration (Figure 3C), while cells elsewhere displayed random movement (data not shown). These results (chemotactic index, migration speed, directional orientation) were similar to those obtained from previous microfluidic chemotaxis chambers [20,21].

## Conclusion

This paper reports a hybrid microfluidic device integrated with vacuum networks and its application in biological experiments. This device provides a simple yet highly adaptable tool for incorporating the advantages of microfluidics to basic biological assays without changing established tissue culture protocols. One of the main advantages of this chamber is that it can be directly used on adherent cells cultured in a standard tissue culture plate. In addition, the hybrid microfluidic-vacuum platform allows for higher throughput, generates stable concentration gradients, and can be useful for studying cellular behaviors of sensitive cells (mouse NPCs, neurons). Using the advantages of this microfluidic platform, we have developed real-time optical assays to investigate cell proliferation and death in mouse NPCs, and chemotaxis of MDA-MB-231 breast cancer cells exposed to EGF gradients.

## Methods

### Fabrication and design of the microfluidic device

The microfluidic device was fabricated in PDMS using previously published soft lithography procedures [18,26-28]. Master molds were made by patterning 100 μm thick negative photoresist (Su-8 50, Microchem, MA) on a Si Wafer. A negative replica of microchannels in PDMS was fabricated by replica molding against the photoresist patterned master mold. The gradient-generating microfluidic network was a simple design with 3 separate inlets (Figure 1A). The main channel was divided into two regions: control and gradient area (Figure 2A). The control region had three separate 200 μm wide microchannels separated by a 100 μm wide barrier. Downstream of the control region, the three channels combined into one channel that formed the gradient region (800 μm wide) (Figure 2A).

### Experimental setup

Sterility was maintained by performing most of the experimental steps, including the assembly process, in a laminar flow bench. Devices sterilized by UV light were assembled on cell culture dishes. Small holes in the Petri dish cover were used to provide vacuum and fluidic channel access (tubing was threaded through these holes), thus maintaining sterility of the device. To avoid bubble formation in fluidic channels, devices were treated with oxygen plasma to make fluidic channel surfaces more hydrophilic. Alternatively, devices were sonicated with deionized water or culture media for 5–10 min before applying to Petri dishes. Before placing the device onto a Petri dish, half of the media was removed by aspiration (approximately 1 ml remaining per 35 mm dish) to facilitate vacuum application. When first assembling the device, vacuum was generated with a 10 ml syringe connected to a 3-way valve and polyethylene tubing with 0.38 mm inner diameter. After placing the device on the microscope or in the incubator, house vacuum was used to maintain continuous vacuum. To monitor cells in real-time, this experimental setup was maintained over 1 day. The Petri dish was placed on a time-lapse microsope with a 37°C, 5% CO_2 _chamber.

### Neural precursor cell (NPC) culture

#### Mice

Wild-type (WT) CD1 mice were used for these studies. To retrieve embryos at certain ages, pregnancies were timed. Successful impregnation occurs at the midpoint of the light-dark cycle (approximately 12 midnight) and is visually detectable the next day by a "vaginal plug", a hardened waxy concretion in the vaginal vault. Noon of the plug date is therefore designated as embryonic day 0.5. All developmental ages were confirmed by embryo crown-rump measurements. All animal studies were done in accordance with Institutional Animal Care and Use Committee guidelines.

#### NPC culture

Telencephalic NPC cultures were prepared from embryos harvested on gestational day 12.5, as described [29]. Briefly, telencephalic vesicles were isolated and placed for 20 min at 37°C in Hanks' Balanced Salt Solution (HBSS, Invitrogen, CA) containing 0.05% trypsin, 0.02% EDTA (Invitrogen, CA), and 0.2% bovine serum albumin (BSA) (ICN Biomedicals, Aurora, Ohio). Trypsinization was stopped by adding an equal volume of 1 mg/ml soybean trypsin inhibitor (Invitrogen, CA) in PBS. Tissues were then mechanically dissociated using several rounds of trituration using a fire-polished Pasteur pipette. Cells were washed once with HBSS containing 0.2% BSA and then resuspended in media with 10 ng/ml fibroblast growth factor 2 (FGF2), 20 ng/ml epidermal growth factor (EGF), and 2 μg/ml heparin. Cells were grown as neurospheres in 6-well non-tissue culture treated plates at the density of 100,000 cells/well at 37°C in a humidified 5% CO_2_/95% air atmosphere for 3 days. Neurospheres were then dissociated with the NeuroCult Chemical Dissociation Kit (Stem Cell Technologies). Dissociated cells were plated on poly-d-lysine and laminin coated 6-well tissue culture treated plates (Corning, NY) at 600,000 cells/well in media with 10 ng/ml FGF2, 20 ng/ml EGF, and 2 μg/ml heparin for 24 hours before applying the microfluidic device.

### Real-time live/dead cell assay

Plated mouse NPCs on Petri dishes were prepared. A microfluidic device was placed onto the Petri dish as described above. Media spiked with PI (2 μg/ml) was applied into the three reservoirs for 30 min prior to exposing with hydrogen peroxide. This provided the baseline for fluorescence intensity of dead cells present at the start of the experiment. After initiating time-lapse fluorescence monitoring, media containing 50 mM hydrogen peroxide was placed into the right reservoir.

### Cancer cell culture

The human metastatic breast cancer cells (MDA-MB-231) were cultured in Leibovitz's L15 media with L-glutamine (Invitrogen, CA) and 10% fetal bovine serum (FBS, Gibco, CA). Cells were plated at a density of 250,000 cells/ml and incubated overnight at 37°C in 60 mm Petri dishes. During the starvation process, serum-free L15 media was added to the cells and then incubated at 37°C for 90 minutes. A microfluidic device was placed onto the Petri dish and attached via vacuum. L15 media with 10% FBS was then added to each of the 3 inlet channels. 50 ng/ml recombinant human EGF (BD Biosciences, MA) mixed with L15 media containing 10% FBS was placed in the right inlet channel. Cell migration was observed for 5 hours using time-lapse microscopy.

### Time-lapse microscopy and migration analysis

The microfluidic device was sealed to adherent cells cultured in a well plate and placed in an inverted microscope (Nikon TE300, NY) equipped with an environmental chamber (37°C and 5% CO_2_). Phase contrast and fluorescence images of cells were taken every 1 min with a digital CCD camera (CoolSNAP cf, Roper Scientific, AZ) controlled with Metamorph (Molecular Devices, PA). Cell tracking and image analysis were performed using Metamorph. Statistical analysis of cell migration speed and angles were performed with Oriana (Kovach Computing Services, UK) using a procedure described previously [20,21].

## Competing interests

The author(s) declare that they have no competing interests.

## Authors' contributions

BGC and JWP developed the hybrid microfluidic-vacuum device and wrote the manuscript. JSH and CH performed the real-time live/dead assay and chemotaxis studies. ESM designed the real-time live/dead assay; mouse work and traditional NPC cultures were performed in his lab. NLJ designed the microfluidic device and finalized this paper; the time-lapse microfluidic culture studies were performed in his lab. All authors read and approved the final manuscript.

## Supplementary Material

Additional file 1 A time-lapse movie of phase contrast images in a control region. A time-lapse movie showing that cells in control regions of the hybrid device grow and divide well. Click here for file

Additional file 2 A time-lapse movie of propidium iodide images in a control region. A fluorescent time-lapse movie shows that cells exposed to hydrogen peroxide die and become labeled with propidium iodide. Click here for file

Additional file 3 A time-lapse movie of phase contrast images in a gradient region. Cells exposed to H_2_O_2 _gradients died from right to left as hydrogen peroxide diffused. Click here for file

Additional file 4 A time-lapse movie of propidium iodide images in a gradient region. An increasing number of cells showed propidium iodide staining over time. Click here for file

Additional file 5 A time-lapse movie of the cancer cell migration in a gradient-generating microfluidic device. Cells exposed to EGF gradients migrate toward higher concentrations of EGF. Click here for file
